# Using the health belief model to identify communication opportunities to prevent Chagas disease in Southern Ecuador

**DOI:** 10.1371/journal.pntd.0006841

**Published:** 2018-09-27

**Authors:** Nelson M. Patterson, Benjamin R. Bates, Amy E. Chadwick, Claudia Nieto-Sanchez, Mario J. Grijalva

**Affiliations:** 1 Infectious and Tropical Disease Institute, Department of Biomedical Sciences, Heritage College of Osteopathic Medicine, Ohio University, Athens, OH, United States of America; 2 International Development Program, Center for International Studies, Ohio University, Athens, OH, United States of America; 3 School of Communication Studies, Scripps College of Communication, Ohio University, Athens, OH, United States of America; 4 Medical Anthropology Unit, Department of Public Health, Institute of Tropical Medicine, Antwerp, Belgium; 5 Center for Research on Health in Latin America, School of Biological Sciences, Pontifical Catholic University of Ecuador, Quito, Ecuador; Common Heritage Foundation, NIGERIA

## Abstract

**Background:**

Chagas disease (CD) is a life-threatening illness caused by the protozoan parasite *Trypanosoma cruzi*, which is transmitted by triatomine bugs. Triatomine bugs inhabit poorly constructed homes that create multiple hiding spots for the bugs. Modifying the actual structure of a home, along with the homeowners’ practices, can reduce triatomine infestation. This research was designed to collect culturally-relevant information to develop a health campaign to decrease risk of CD transmission by promoting home maintenance and better hygiene in rural communities of southern Ecuador.

**Methods and main findings:**

The Health Belief Model (HBM) guided focus group discussions and the interpretation of the results. Four focus groups ranging from 4 to 10 participants were conducted between May and June 2014 in three communities of Loja province in Southern Ecuador. A thematic analysis was used to identify within the data related to perceptions of susceptibility, severity, benefits, barriers and self-efficacy related to CD and its prevention. The results provide clear guidance for the development of Chagas-prevention messages.

**Conclusion:**

Data obtained emphasize the importance of standardizing messages presented to the communities for CD prevention. Messages should provide more information on the protective nature of the behaviors promoted for CD prevention; overcoming barriers such as cost and convenience, and build on facilitating factors, including community members’ interest on quality of life, protection of their families, and relationship with the land.

## Author summary

This study focuses on Chagas disease (CD) prevention in southern Ecuador. This region has a high rate of triatomine infestation. We used the Health Belief Model (HBM) to understand why people do or do not engage in CD preventive behaviors, particularly those related to home improvement. Additionally, we wanted to learn how to communicate most effectively about CD prevention. We gathered information about community member’s perceptions of CD in four focus groups, with a total of 26 participants. Our results indicate that communities do not see triatomines and CD as a threat. Lack of structures, cost, and convenience were the three key barriers mentioned by research participants for implementing CD preventive behaviors. However, participants were open to work with prevention programs since they saw it as an opportunity for community members to work together under the motivation of improving their living conditions. Capitalizing on motivators and removing barriers will be important for subsequent communication campaigns. Messages evoking fear of triatomine bugs and CD will contradict participants’ personal experience; instead, messages focusing how these behaviors will improve communities’ quality of life, protect their families, and strengthen their relationship with the land, will be more acceptable and appealing.

## Introduction

Chagas disease (CD) is a life- threatening illness that affects 7 to 10 million of people around the world. The disease is caused by the protozoan parasite *Trypanosoma cruzi* [1]. The main vectors for *T*. *cruzi* transmission are triatomine insects, nocturnal blood-sucking insects mainly found in the southern United States and in most of Central and South America. Currently, there are 140 species recognized in the triatomine subfamily [2].

CD is a leading cause of deaths and lost disability-adjusted life years (DALYs) from neglected tropical diseases NTDs in Latin America; it is the most important parasitic disease in the region [3, 4]. Up to 10 million people worldwide may be infected with *T*. *cruzi* [5]. There are approximately 300,000 cases of CD in the United States, in addition to thousands of cases documented in Canada, Australia, Japan and several countries in Europe. Global cases are increasing because of global migration patterns [4].

Currently, there are limited treatment options for CD, making prevention of paramount importance. Pesticides have been used in and around infested homes to combat triatomines. Government and large NGOs have used large-scale, top down approaches to control effectively vectors, but pesticide spraying is not a long-term or sustainable solution [6, 7].

To understand factors that affect home-related behaviors and guide intervention efforts, our research group operates programs in three rural communities in the southern province of Loja, a province in Ecuador’s inter-Andean valley of [8]. Ecuador has approximately 200,000 people affected by CD, and millions more are at risk of being exposed [9]. The Healthy Living Initiative (HLI), a program initiated by the Infectious and Tropical Disease Institute at Ohio University and the Center for Research on Health in Latin America (CISeAL) at Pontifical Catholic University of Ecuador, has been working in rural communities in Southern Ecuador since 2010 developing a sustainable model for CD prevention based on community participation and home infrastructure improvement. The main component of HLI is Healthy Homes for Healthy Living (HHHL), a program specifically designed to create healthy environments leading to healthier lifestyles with particular emphasis on CD prevention.

Since 2010, HHHL interventions include infrastructure improvement and promoting preventive practices. A total of 46 practices were identified in a positive deviance study [10]. To encourage community members to participate in future home improvements and Chagas prevention activities, HHHL needs to know how to communicate these prevention behaviors effectively. Using the Health Belief Model constructs as theoretical guide, this research aims to understand factors that predict the adoption of CD prevention practices promoted by HLI.

### Chagas, a neglected tropical disease

The World Health Organization categorizes CD as a neglected tropical disease (NTD)[11]. Chagas and other neglected tropical diseases are poverty-habilitated issues also derived from insufficient access to drinking water, sanitation, inadequate housing, education and health services [12–15].

One key to long-term CD prevention is to prevent infestation of the home. Inadequate housing is associated with higher infestation [12]. Physical barriers between the home and surrounding environments can prevent triatomines from populating the home [16]. These insects enter through and live in the cracks of poorly constructed homes in rural areas; dirt floors, thatched roofs, and exposed eaves also provide entry points. Housing improvements can lower the infestation rate of the insect and increase the quality of life of individuals [17].

Although issues of home construction are significant [8, 10], practices in and around the home (the peridomicile) also increase the risk of infestation [12, 18]. Field observations and prior research indicate that in poorer houses, with lower incomes and fewer residents with formal education, community members perform behaviors that create suitable conditions for triatomine infestation [8, 12]. The strongest four determinants of triatomine infestation are the number of dogs allowed to enter the home [19], having chickens in a corral or not [8, 9, 18], cleaning of trash from the peridomicile or not [9, 11], and being located in the boundary of the village or not [12, 18]. Firewood and rock piles also can increase risk of infestation [8, 19]. Promoting healthier housing practices will create a sustainable solution for triatomine prevention, lowering exposure to CD [6, 7]. To protect adequately humans from contracting CD there is a need to effectively communicate the risk and propose viable preventive measures, particularly the control of triatomine vectors [20].

### Health belief model

The health belief model (HBM) provides a framework for this kind of communication. The HBM can be used to predict preventive health behaviors and to develop interventions [21, 22]. This model has been used extensively in multiple health contexts [23–25]. The HBM is comprised of several constructs that, in combination, predict behavior. They are: perceived threat (comprised of perceived susceptibility [an individual’s assessment of the likelihood of a negative health condition occurring] and perceived severity [an individual’s assessment of the seriousness of contracting the health condition and its consequences]); likelihood of action (the weighing of perceived benefits [rewards attributed to engaging in the recommended behavior] against perceived barriers [obstacles that deter an individual from executing the recommended behavior change]; cues to action (motivating factor that provokes or encourages change); and, self-efficacy (one’s belief in one’s ability to actually perform (and maintain) the desired behavior change).

The HBM guided our investigation of factors affecting the adoption of CD prevention behaviors. Specifically, we asked the following research questions,

**RQ1**: What is the perceived threat (perceived severity and perceived susceptibility) of triatomine infestation among community members?

**RQ2**: What factors affect the likelihood of action (perceived barriers and perceived benefits) to prevent triatomine infestation among community members?

**RQ3:** What level of self-efficacy for triatomine control is expressed in these communities?

**RQ4:** What cues to action for triatomine control do community members recall?

## Methods

### Ethics statement

Ohio University Institutional Review Board reviewed and approved the protocol, including administration of oral informed consent, all recruitment materials and data collection procedures involving human participants (OU IRB 14-E-158). All human subjects were adults. Oral informed consent was collected in this case because the research is minimal risk and cultural norms make participants wary of signing documents.

### Study site

This study took place in Loja province, Ecuador, which has a high rate of triatomine infestation and, consequently, a high risk for CD presence [8, 26, 27]. Since 2010, HLI has collaborated with three adjacent rural communities–Guara, Chaquizhca and Bellamaria–in this province (5). These three communities were selected as pilot communities for HLI because of the high rate of infestation of triatomines [8, 10]. All three communities have similar characteristics, with most of the inhabitants participating in subsistence farming. The three communities comprise ~150 family homes spread through mountainous terrain in Ecuador’s southern highlands. These communities face limited access to clean water and sanitation facilities, along with insufficient transportation to reach the larger nearby town of Cariamanga, the main commerce point in this area. The homes are far apart. Community members often must walk one or two hours to get to the main road to find transportation to go to the city. Most of the homes in these communities–excepting those (re)constructed in HLI interventions–have structural, behavioral, and peridomiciliary conditions associated with triatomine infestation.

### Participants

Focus groups were conducted to identify community perceptions of CD and their views of healthy practices identified by HLI as important. All male and female adults (over age 18) who lived in the communities of Guara, Chaquizhca and Bellamaria were invited to participate. The first author and a field community worker made home visits and spoke with community members at local gathering points to ask for participation in the focus groups. After three weeks of recruitment, meeting dates and locations were arranged with potential participants. Men and women were separated for most of the focus groups to promote open dialogue [28]; however, a mixed focus group was also conducted to allow us to assess the possible influence of gender roles.

Four focus group discussions were held. A mixed group, with five women and four men, met in the Bellamaria community center. A second group, also in the community center, consisted of four women. A focus group in Guara, comprised of nine women, and a focus group in Chaquizhca, comprised of four men, were held in the communities’ respective elementary schools.

### Procedures

Prior to conducting focus groups in the selected communities, a moderator guide was designed to systematically incorporate the HBM constructs and to encourage dialog between group members. Following initial construction, we consulted with HHHL staff that work in the communities on a daily basis [28] to modify the guide. This allowed us to employ terms and expressions used by community members. There was also a practice focus group conducted with HHHL staff to consolidate the discussion and to adjust the ordering of questions, as well as to assess the natural flow of conversation.

The focus group team consisted of a moderator, note taker, and assistant. The focus group moderator conducted the actual focus groups. The note taker made observational notes of the groups and individuals expressions. The note taker also wrote down the participants’ comments about the behavior that were easy/hard. The assistant helped arrange the room prior to the focus groups and helped explain the task of identify behaviors as easy/hard. The room was set up with chairs in a circle allowing all participants and the moderator to look at one another.

Upon arrival, participants were told the purpose of the research. They were then asked to provide individual oral informed consent. A short icebreaking activity followed. Participants stated their names and described the part of their homes they liked most. This question helped build comfort in talking about their homes and to build good rapport. The question also assisted the transcriptionist in identifying the voices of the participants. Following the icebreaker, the moderator started the official focus group.

The focus group discussions followed constructs of the HBM. The first section asked participants to explain what they knew about CD and triatomines. Next, the participants viewed an educational video. The educational video highlighted the pathogenesis of CD, the transmission via triatomines and then described behavior changes that can help prevent triatomine infestation. Following the videos, participants contributed to an interactive exercise in which they rated behavior changes listed in the video as easy to execute, hard to execute, or neither. Participants placed different color sticky notes on poster boards to represent each behavior as “easy”, “hard”, or neither. The activity enabled participants to move around the room and discuss the behaviors with one another. Following the participatory activity, the participants discussed why they chose to answer the way they did, and their responses were written on the poster board by the note taker. The participants concluded by providing suggestions for promoting the behavioral practices in their communities, including possible media and channels.

### Analysis

The focus group data were analyzed, verified, and reported. The first author transcribed the focus groups verbatim in Spanish; then the transcripts were translated into English for reporting. A thematic analysis was conducted to find information useful for designing a health campaign [29]. Commonalities in the focus group dialogs were identified as themes; these themes were assigned specific codes following Faraday and Muir-Cochran’s stages of coding method [30]. The data were coded through MAXQDA-10. An initial coding manual was developed. Two bilingual researchers then coded the transcripts and compared notes for reliability. The first author developed the initial codes; the fourth author used the same codes to test their reliability. The fourth author also added her own codes as needed. The two coders discussed the codes before analyzing all the focus groups using the common codes for a second time. The codes were then summarized under Health Belief Model constructs for reporting.

## Results

Our analysis applied the health belief model constructs to examine perceptions that would facilitate or interfere with a successful Chagas vector control program in these communities. Overall, triatomine vectors are seen as a low threat to health. Likelihood of action is enabled by beliefs that triatomine control results in a clean home, but disabled by beliefs about the cost and convenience of controlling these vectors as well as beliefs that the benefit can be attained through pesticide use. Members of the community rarely indicated a sense of self-efficacy, and they expressed little awareness of cues to action other than those offered by the Healthy Living Initiative.

### Perceived threat

The first research question asked what the perceived threat of triatomine infestations was in these communities. The perceived threat for adults was low because, although members of the community believed they were susceptible to insect bites, the consequences of these bites were minimal. Threats to children were seen as higher because children were viewed as suffering more severe consequences.

In each focus group, the facilitator asked about the possibility of community members being bitten by triatomines and whether they thought CD was present in their communities. Few participants mentioned knowing individuals affected by the disease in their communities. One man from the Bellamaria group stated:

I have heard that in Chaquizhca there is someone with the disease and they have to travel far to get treatment so they can be protected. I don’t know if it will cure them or if they will continue to be sick because the disease seems to be somewhat complicated.

Some participants mentioned that there were fewer triatomines in their communities than in other communities in the country. One woman, also from Bellamaria, stated, “It seems that in other regions there are more [triatomines] than there are in our neighborhood Bellamaria because other places are cooler.” Other participants, in other focus groups, agreed that because of the warmer climate in Loja there were lower rates of infection.

One theme consistent in all focus groups was that being exposed to triatomines was not associated with an elevated risk of contracting CD. Triatomines were recognized as being present in the participant’s homes; however; many participants stated that the triatomine infestation had decreased in recent years due to the HLI project. One female participant from the mixed focus group expressed her view of triatomine bugs, saying:

I know they are little bugs that [people say] are dangerous… before, there were a lot of bugs around the children. They used to be on the walls and sometimes they came out to bite us.

This woman noted that the triatomines have been in her home and had bitten her and her family, but she did not mention any ill health effects. Other community members agreed, as they too had experienced similar interactions with the triatomines. Her statement, and those who agreed with her, reflects a belief that susceptibility to a bug bite is high, but the consequences of the bug bite are not severe. Because threat is a combination of both severity and susceptibility, perceived threat in these communities may be low.

Even after viewing the video with information about being exposed to triatomines, participants did not indicate a high threat of Chagas disease from the bugs. In the men’s focus group and the mixed focus group, lack of connection between vectors and disease was also present. Participants expressed little concern about their own risk of contracting CD. Other participants held similar sentiments when discussing past interactions with the triatomines from both male and female groups. The focus group moderator probed about the perceptions held by the larger community. One woman summarized the communities feelings well: “Almost everyone knows about it [CD] but nobody takes it seriously”.

Although adults did not feel a threat from triatomines or CD, they did feel that their children were at risk. In the women’s group in Bellamaria and the women’s group in Guara, participants felt that there was a higher possibility of their family being affected by CD. Many of the women’s comments argued that triatomine bugs could harm their children. As one woman said, “We need to be very cautious and think about the kids, because it is serious… I know they have bitten us and it’s not life threatening for us but for our children”.

One woman from Bellamaria shared her personal story of how triatomines could hurt children. She said that her brother died of CD. She stated that her brother lived with their family until fifteen years ago, when he moved to the capital city, Quito. In Quito, her brother started having symptoms and received treatment. She expressed that, when they were younger, they were likely to have been bitten by triatomines. She was not sure whether her brother contracted CD, but she thought his early life in Bellamaria was why he contracted this illness.

Men were also concerned about their children’s risks. They mentioned that children are more susceptible to contracting CD than adults. A male participant from Bellamaria stated, “We need to be really careful and think about the children, as it is very serious for them.” He described having seen triatomines in his community. He did not state anything about being at risk himself, only his children. Another man, from the Guara group, stated, “When a chinchorro bites a person, at least when it bites a child, it affects it more because the body reacts in a bad way like they said in the video, where the heart gets bigger.” The majority of participants expressed these sentiments both before and after the educational video. Thus, even if there is little personal threat perceived by adults, there are threats they perceive to the younger members of their communities.

### Likelihood of action

The second research question asked what factors in the community affected the likelihood of action. Perceived barriers to action were mainly issues of cost and lack of storage for animals and material. The overarching barrier is these communities believe they have few economic resources to allow them to enact the desired changes. The perceived benefits of controlling triatomines were a sense of pride in the cleanliness of homes and the ability to protect children, yet these benefits were also seen as attainable through pesticide spraying.

#### Perceived barriers

One salient barrier for participants was the lack of resources in their communities. Participants did not perform some of the recommended behaviors because of cost. The addition of screen doors and windows is a way to improve the physical barriers between homes and outdoor environments. Adding screen doors and windows were not perceived as difficult, but the mesh used to make the screen doors is seen as costly. One woman stated, “The mesh that [is used] for making these doors is expensive, in Cariamanga it costs $5 per meter.” Both male and female participants expressed their concerns about the maintenance of these new screens. If there was a tear in the screen caused by normal use, they were not sure whether they could afford to have the screens repaired or replaced. Other alternatives to screen doors and windows were not discussed by the participants.

In addition to cost, recommended behaviors like storing excess materials outside the home or penning animals where they did not contact the walls of the home were seen as difficult to perform. When participants were asked to give their opinion about separating farming and building materials from the home, most participants responded that they would like a specific location where they could safely store these materials without risk of theft or damage, but that they did not and could not do so. One woman from the Guara said, “It is not easy because we need a place where we can put everything so it doesn’t get destroyed by the water and sun.” Participants in all focus groups stated their desires to have a shed where they could store these materials. When asked whether there were other options for keeping these materials away from the home without structural additions, the participants did not come up with any alternatives. One participant from Chaquizhca stated that, in their home, he has one room or a corner of a room where he would keep such materials. This method was common for other participants from all of the focus groups. One participant commented, “I always place [materials] in a corner when it is something that still has some value and when it doesn’t [have value], I get rid of it by burning it.” This action, however, means that triatomines have additional places to hide in his home.

In addition to spare materials, community members often keep firewood in or beside their homes. Many participants stated that they now have incorporated gas stoves for daily cooking, but there are traditional dishes for which they still use firewood. A small portion of the participants also stated that they still use firewood as their main means of cooking. When asked about the storage of firewood, the majority of participants stated that they kept the firewood next to the home. However, during the rainy season, participants would transfer the wood into the home. The firewood was kept inside to keep the wood dry. It was unclear how much firewood participants kept inside the home; however, some stated that they kept less than a week’s supply of firewood in their homes. One participant in the men’s focus group in Chaquizcha stated, “We bring a bundle of wood on our donkey and with that we have about four days of firewood for the house.” These practices create additional barrier to change because they provide additional places for triatomines to nest and facilitate carrying insects from outside into the home.

Similarly, participants mentioned lacking structures for proper food storage. Most participants stated that they possessed a refrigerator in their home where they could keep cold or perishable food safely. The few participants who did not have a refrigerator stored food products in their kitchen with no means of preserving food items. Many participants reported having no proper storage for bulk products like corn, peanuts, and beans, however. Participants reported storing these products in burlaps sacks, where they are more susceptible to rodents and could affect the sanitation of the home. If rodents are present close to the home, they provide triatomines with another entry into the home.

In relationship to animal husbandry, all participants expressed that it was not desirable to have chickens confined in chicken coops. They stated that when chickens are kept in a coop, the chickens do not develop as well as chickens that were free ranging. The participants stated that when the chickens were confined, they are more susceptible to diseases. Confined chickens are also perceived as more likely to become injured from fighting amongst. Participants also commented that when chickens are not confined, they are able to forage for their own food, which meant that the community members would not need to buy extra food for their chickens. However, when participants were asked about solutions for keeping chickens away from the home, many mentioned the construction of an enclosed area. Nonetheless, the hens nests are often been kept against the sides of exterior home walls for protection from rain and wind. When asked about hen nesting practices, the participants had varied customs based on their own family or traditional experience for caring for hens. Some stated that they changed the nest every few weeks, while others said they changed the nests every two to three months. One woman from Guara stated that she cleans the nest and then sets them out to dry to reuse them. All of these practices raise the likelihood of home infestation. Similarly, with larger animals like goats, participants stated that, the more the animals are able to forage for their own food, the less the owners need to provide food for them. And, by keeping these animals closer to the home, the participants felt the animals would be safer. Thus, penning was not a preferred means for animal care, and few barrier to their coming near the home were erected.

The exception to allowing animals to forage freely was guinea pigs. Participants mentioned that they buy supplemental food products for these animals. Guinea pigs traditionally are kept inside the home to protect them from predators. According to the participants who had guinea pigs, the animals like the heat that is produced in the kitchen while cooking and this was another reason they kept the guinea pigs inside. A male participant from the mixed focus group stated, “They are a little delicate because if you remove them from the kitchen, they will die. They like the temperature from the heat and ashes.” However, in all focus groups, there were participants who stated that they no longer had guinea pigs because they would leave a foul odor and they were too time-consuming to care for.

#### Perceived benefits

Participants perceived the main benefit of preventing infestation to be pride in their homes. Participants were proud of their ability to keep a clean home despite their lack of resources. One male participant explained, “It doesn’t matter how poor we are, it is important to have a clean home, it is a little harder because we are poor but we try to keep things clean”.

This benefit was challenged, however, in that the community members believed they could attain the benefit by using pesticides. All community members reported using pesticides for controlling infestation in and around their homes. Therefore, they did not see the need for the prevention behaviors. When asked about controlling rodents in their homes, one participant in the male focus group stated that, “When laying out the poison for the rats, one has to put it out in the afternoon. Then in the morning you have to remove it because a chicken could come and eat it, killing it quickly.” Others stated that they used insecticides every two to three months for general maintenance from mosquitos and other common insects in their homes; however, none of the participants mentioned using fumigation for preventing triatomines or CD.

### Self-efficacy

The third research questions asked what the level of self-efficacy expressed in the communities was. Participants rarely expressed statements related to self-efficacy. They did not discuss confidence or lack of confidence in several skills related to CD prevention, such as ability to identify triatomines or to trap the bugs. In one of the few moments where self-efficacy was discussed, when asked what happens if they see the triatomines in their homes, one woman stated, “Now I catch the bugs in a plastic bag or grab them with toilet paper.” Other participants stated that they simply kill the triatomines in their homes, but there was little depth of discussion.

### Cues to action

The final research question asked what cues to action for triatomine control the community members recalled. Participants identified few cues to action or reminders to engage in healthy home behaviors.

Although assessing the effects of previous HLI efforts was not a focus of this research, participants clearly indicated that most of their knowledge about CD and triatomines was due to the efforts of our research and intervention group. Specifically, identifying different species of the triatomines and their different stages of development was connected to the educational calendars that were distributed to community members in 2012. Participants from the focus groups stated that the majority of the information they possessed on CD originated from the interaction with the health workers who fumigated their homes and the calendar. A few community members stated that they like to decorate their homes with pictures and memorabilia and the calendars added to their decor.

We also asked whether the community needed a communication campaign to help educate and motivate community members. They were receptive to the development of a health campaign, suggesting it would be beneficial for others in their communities to have access to the educational video that they watched during the focus groups. After viewing the informational video, one participant from the mixed focus group stated: “I know that some people [in the community] lack some of this information, but with these videos that’s when people start to become concerned with their health and the health of their families”.

Participants from all focus groups expressed that it was important for the communities to collaborate in efforts to improve their living conditions. When prompted about communication channels, all of the participants stated that they used cell phones more than any other communication means. Following cell phones, face-to-face conversations were a common communication channel. Television and radio were used rarely because some participants did not have the electronic devices in their homes. The participants who stated that they used radio or television also mentioned that reception was precarious.

## Discussion

The purpose of this research was to gather the perspectives of the community members from Guara, Chaquizhca and Bellamaria to inform communication campaigns to support reducing the risk of triatomine infestation and, thus, the risk of CD. We used the HBM to understand factors that affect CD prevention behaviors. Each of the HBM constructs offers insight into such a campaign. For each construct, we summarize the findings and provide recommendations for communication campaigns.

Changing how communicators frame the threat of Chagas disease may be important. Overall, participants had low perceptions of severity and susceptibility for CD. In general, participants felt susceptible to being bitten by triatomine bugs, but did not feel particularly susceptible to Chagas disease. Because participants did not feel threatened by CD, they were not motivated to change their behaviors to protect themselves. They did see higher threat toward children. Therefore, to increase perceptions of severity and susceptibility, messages could focus on the triatomines’ ability to affect family members, particularly children. Although the transmission occurs via the triatomine feces, messages should clearly link being bitten by triatomines to higher risk of contracting CD. To further reinforce preventive behavioral practices, health projects can focus on enhancing perceptions of severity by outlining how there is no medical treatment readily available in this region for CD if a child is bitten [9].

Likelihood to change is disabled by the barriers identified by participants. A major barrier perceived by community members dealt with the direct and indirect costs of preventive behaviors. Many community residents do not have sufficient income to invest directly in their homes and this was the reasoning given by participants for not performing protective practices, such as building special storage areas or buying window screens. Added costs for animal husbandry also serve as barriers to change. Because constructing storage areas and reconstructing the home provides many benefits, communication campaigns should not only recommend home renovation but also provide supporting resources and skills training to help participants overcome financial barriers. In addition, to overcome barriers, health campaigns should emphasize activities that do not require significant resources; low cost techniques that are easily accessible, such as daily cleaning and separation of animals and humans, have lower barriers to performance.

The participants’ responses also indicate several factors that should be emphasized to support likelihood to change. Overall, collaboration between HLI and the community members was perceived to be a positive. In some cases, community members perceived having a home they could be proud of to be a key benefit of, and motivator for, the recommended prevention behaviors. Future interventions should continue to understand community needs and associate as many benefits of triatomine control as possible. Campaigns need to clearly demonstrate the value of the campaign and the value of the behavior for the community based on what the community values. For example, preventing Chagas disease may not be a goal for community members, but improving quality of life is. Community members also express pride in their homes, land, and animals. Communication campaigns should recognize and appeal to this pride as a motivator for behavior change.

Pride in one’s home as a pathway toward action raises a dilemma for campaign designers. Articulating the importance of cleaning and maintaining one’s home without offending individuals by implying that their homes are “dirty” is difficult. Because most campaign designers are “outsiders,” it is particularly important to sustain positive relationships between the researchers and the community. Prevention messages should not focus on the negative aspects of having an unkempt home, but on the positive outcome of protecting one’s home and family against insects. The stigmatization associated with having a disorganized or dirty home could have a negative impact on individuals and their relationship within the community, as well as with prevention programs.

Community members recalled few cues to action, or reminders for them to engage in CD prevention behaviors. One exception was the calendar that HLI provided that showed different types and phases of triatomines. Interventions should consider including prevention behavior messages in materials that are both decorative and useful like the calendar. Beyond the calendar, members of the community suggested channels for future communication interventions. Future interventions should consider using mobile technology to communicate messages, as communication among community members is mainly via cellphones. Further projects should consider using mobile health technologies as tools to disseminate information about Chagas as well as other health problems.

Community members’ self-efficacy for recognizing and killing or removing triatomines is unclear. It is positive that community members know characteristics of triatomines and associate the insects with harm to them or their families. Communication materials should build on this familiarity with triatomines. Therefore, interventions should seek to build on already existent precursors for self-efficacy to assist community members in building self-efficacy for related behaviors.

### Limitations

This research was conducted in small rural communities (152 homes total), mainly populated by families relying on agriculture-based activities. Local families are usually busy in their fields, which limit participation in non-essential activities. The number of focus groups conducted was sufficient for the small size of these communities; however, the participants who had sufficient time to participate may have differed from other members in the community who did not have this time. In addition, because these three communities have been exposed to previous HLI interventions, they may be more aware of triatomines and CD than other similarly situated communities in Loja province. Finally, although the focus group moderator was fluent in Spanish, some local colloquial words may have been lost in translation.

### Conclusion

This article used the health belief model to examine factors that predict behaviors associated with decreased triatomine infestation, and thus decreased Chagas disease transmission, in rural Ecuador. Neglected tropical diseases such as CD are difficult diseases for which to design communication campaign because there are few visible symptoms and consequences. Understanding perceptions of community members in terms of threat, likelihood of action, self-efficacy, and cues to action may enhance prevention efforts. To identify best practices, further research is needed on best health campaign practices in the context of communities that are at risk of neglected tropical diseases. A key element to decreasing exposure for CD and other NTD, comes from designing campaigns that help communities adopt preventive behaviors most fitting for them.
